# Biofabrication of chitosan/chitosan nanoparticles/polycaprolactone transparent membrane for corneal endothelial tissue engineering

**DOI:** 10.1038/s41598-021-86340-w

**Published:** 2021-03-29

**Authors:** Tahereh Tayebi, Alireza Baradaran-Rafii, Abbas Hajifathali, Azam Rahimpour, Hakimeh Zali, Alireza Shaabani, Hassan Niknejad

**Affiliations:** 1 Department of Tissue Engineering and Applied Cell Sciences, School of Advanced Technologies in Medicine, Shahid Beheshti University of Medical Sciences, Tehran, Iran; 2 Ophthalmic Research Center, Department of Ophthalmology, Shahid Labbafinejad Medical Center, Shahid Beheshti University of Medical Sciences, Tehran, Iran; 3 Hematopoietic Stem Cell Research Center, Shahid Beheshti University of Medical Sciences, Tehran, Iran; 4 Department of Polymer and Materials Chemistry, Faculty of Chemistry and Petroleum Science, Shahid Beheshti University, Tehran, Iran; 5 Department of Pharmacology, School of Medicine, Shahid Beheshti University of Medical Sciences, Tehran, Iran

**Keywords:** Biotechnology, Biomaterials, Biomaterials - cells

## Abstract

We aimed to construct a biodegradable transparent scaffold for culturing corneal endothelial cells by incorporating chitosan nanoparticles (CSNPs) into chitosan/polycaprolactone (PCL) membranes. Various ratios of CSNP/PCL were prepared in the presence of constant concentration of chitosan and the films were constructed by solvent casting method. Scaffold properties including transparency, surface wettability, FTIR, and biocompatibility were examined. SEM imaging, H&E staining, and cell count were performed to investigate the HCECs adhesion. The phenotypic maintenance of the cells during culture was investigated by flow cytometry. Transparency and surface wettability improved by increasing the CSNP/PCL ratio. The CSNP/PCL 50/25, which has the lowest WCA, showed comparable transparency with human acellular corneal stroma. The scaffold was not cytotoxic and promoted the HCECs proliferation as evaluated by MTT assay. Cell counting, flow cytometry, SEM, and H&E results showed appropriate attachment of HCECs to the scaffold which formed a compact monolayer. The developed scaffold seems to be suitable for use in corneal endothelial regeneration in terms of transparency and biocompatibility.

## Introduction

Cornea, the anterior transparent part of the eye, has a critical function for vision by transmission and refraction of the light into the eye as well as protecting the internal portions of the eye from external damages. Anatomically, the cornea consists of three layers from the outside to the inside including the epithelium, the stroma, and the endothelium^1^. Damage to each layer can cause serious visual impairments. The hydration degree of corneal stroma is the main determinant of corneal clarity which is regulated by dual pump and barrier roles of the corneal endothelium^2^. Human corneal endothelial cells (HCECs) do not proliferate in vivo and the cell loss to below the critical density caused by different condition such as aging, corneal disorders, and physical and chemical trauma lead to the visual impairments^3^. Full-thickness penetrating keratoplasty (PK), endothelial keratoplasty (EK), and two modern selective EK, Descemet's stripping automated endothelial keratoplasty (DESAK) and Descemet's membrane endothelial keratoplasty (DMEK) are generally used as the therapeutic choices for endothelium injuries^4,5^. However, these methods have faced many problems such as universal donor shortage and transplant failure due to post-surgical inflammation^6^. Hence, it seems necessary to find alternative therapeutic approaches to surmount these undesirable limitations.

Regenerative medicine strategies, including cell/tissue therapy and tissue engineering, have opened new windows toward corneal endothelial regeneration^7^. Several scaffolds have been studied for cultivation and transplantation of human corneal endothelial cells^8^. Essentially, the scaffolds should provide a biochemically and biomechanically proper microenvironment for cell behaviors such as adhesion and proliferation^9^. Furthermore, a suitable corneal scaffold should have other important characteristics such as biocompatibility, biodegradability, and transparency^10^, as well as does not stimulate immune responses.

Both natural and synthetic polymers are used to engineer the corneal scaffolds. Gelatin, collagen. silk, hyaluronic acid, cellulose, and chitosan are natural polymers that were commonly used for corneal tissue engineering. Natural polymers show appropriate biological properties such as biocompatibility and biodegradability and are suitable for application in biological approaches^9,11^. Chitosan is one of the most outstanding polysaccharides that candidates for application in scaffolding and is broadly used in tissue engineering, pharmaceutical and food industries due to its exclusive biological and biochemical properties^12^. Aqueous solubility, biocompatibility, biodegradability, antibacterial and antifungal activity, mucoadhesive and homeostatic features make chitosan as a good basis for cell attachment and growth^13,14^. Although, chitosan films are formed easily by the solvent casting and subsequent evaporation of the solvent^15^, the films show poor mechanical properties, and are easily broke down^16^. Poor mechanical property is the main drawback of chitosan and some other natural biomaterials which limits their application in designing the scaffold. Incorporating nanoparticles into scaffolds is a suitable method to overcome this limitation. Homogeneous dispersing the nanostructures in the chitosan background improves mechanical properties of the matrix^17,18^. Chitosan nanoparticles (CSNPs) have also attracted great interest in the scaffolding due to their specific biological effects. Studies have shown that CSNPs have a greater antimicrobial effects compared with chitosan^19,20^. CSNPs have a large surface-to-volume ratio which provides a great binding capacity for biological macromolecules which is an important factor for controlled releasing of drugs or growth factors^21^. As well, this property improves the mechanical properties, surface energy and surface reactivity of the scaffold which results in more cell-scaffold interactions^22^. In addition, incorporation of CSNPs in a scaffold provides an opportunity for loading the growth factors and signaling molecules into the scaffolds which would be helpful to improve cellular function on the scaffold through controlled release^23^.

Improvement of the biomechanical properties of the chitosan films will be achieved by the other modifications such as plasma surface treatment^24^, irradiation^25^, and incorporation of other material particles or mixing with other polymers^26^. Poly-Ɛ-caprolactone (PCL), is a FDA-approved synthetic polymer that is extensively used in regenerative medicine. Easy processing capability and susceptibility of surface modifications make PCL as a suitable choice for biomedical aims such as eye tissue engineering and drug delivery^27,28^. In addition, incorporation of PCL in the scaffold caused structural integrity of scaffolds designed for ocular purposes^28^.

Combining the polymers results in production of a wide range of composite scaffolds with desirable biological and biochemical characteristics (i.e. improved biocompatibility and hydrophilicity) which could enhance the corneal regeneration^27,29,30^. So, the aim of the present study was to design and construct for the first time a biodegradable and transparent scaffold as a suitable carrier for corneal endothelial cells by incorporating chitosan nanoparticles into the PCL/Chitosan composite. Therefore, we prepared several CSNPs/PCL/CS composite films with different proportions and examined clarity, surface wettability, toxicity, and cell and surface interaction to obtain an appropriate composite scaffold for culturing HCECs and application in corneal endothelial tissue engineering.

## Materials and methods

### Preparation and characterization of chitosan nanoparticles

Ionotropic gelation method was used to produce the chitosan nanoparticles (CSNPs). At first, 0.9 mg/ml solution was prepared by dissolving the medium molecular weight chitosan (75–85% Deacetylated, Sigma Aldrich) in acetic acid (0.5% w/v) under magnetic stirring for 24 h. The pH of solution was adjusted on 5.5 using NaOH (1 M) and filtered by 0.45 µm acetate cellulose filters. Sodium three-polyphosphate (TPP) was dissolved in deionized water (0.25 mg/ml) and filtered with 0.22 µm acetate cellulose filters. 2 ml TPP was added drop wisely to each 5 ml chitosan solution while allowed to stir. After that, the nanoparticle solution was centrifuged at 27,670*g* for 1 h and the supernatant was discarded. Finally, the CSNPs were obtained after freeze-drying for 48 h.

The size and PDI (Polydispersity Index) of CSNPs were measured by dynamic light scattering (DLS) technique (Cordouan Technologies, NanoQ V2.5.9.0, France) for purified CSNPs. For this purpose, freeze-dried CSNPs were suspended in 0.5% acetic acid and sonicated in a sonication bath (Ultra, VGT-1860QTD, Korea). Then, the DLS test was carried out. Furthermore, the morphology of CSNPs was evaluated with scanning electron microscopy (SEM).

### Construction of chitosan/chitosan nanoparticles/polycaprolactone scaffolds

PCL (Sigma Aldrich, Mn 45000 by GPC) solutions were prepared at three different concentrations of 0.1%, 0.15%, and 0.2%, in acetic acid. CSNPs (1% w/v) were also dispersed in acetic acid 0.5 M and sonicated. Chitosan dissolved in acetic acid 0.5 M to achieve 1% (w/v) solutions. In order to make a gelled matrix for other components, a constant concentration of chitosan (25% w/w of final scaffold) was considered for all scaffolds by adding the 1 ml of chitosan solution drop wisely to 10 ml of each PCL solutions under gentle magnetic stirring. Since, solubility of CSNPs in acetic acid decreases due to cross-linking with TPP appropriate volume of 1% sonicated CSNPs suspension were added to 10 ml of 0.1%, 0.15% and 0.2% PCL solutions, to achieve the mixture of 50%, 37.5%, and 25% CSNPs, respectively. After 2 h stirring, the equal volumes of each solution were cast into the silicon plates with the same diameter and depth followed by drying in an oven at 55 °C under an unsaturated humidity state. To investigate possible changes in the size of CSNPs in the final scaffold composition, DLS analysis was performed before casting the composite solution. In all experiments, the scaffolds were cross-linked using 0.2% glutaraldehyde for 30 s before any application.

### Surface wettability

The surface wettability of composite scaffolds was assessed via the measurement of water contact angle (WCA) using a contact-angle instrument (OCA PLUS 15, Data-physics, Germany). For this purpose, the average WCA value of both sides of the scaffold was measured after putting deionized water droplet. This experiment was done three times for each composite membrane.

### Evaluation of transparency

The light transmittance property of developed scaffolds was evaluated at 450 nm and 600 nm wavelengths using a UV-spectrophotometer (Cecil, United Kingdom). All specimens were hydrated with PBS and flattened between two coverslips without shrinkage and air bubbles. Then, the spectrophotometric assay was performed triplicate to measure the percentage of light transmission in both wavelengths. Two coverslips moisturized with PBS were used as blanks. The cadaver acellular corneal stroma obtained from EYE Bank of I.R. Iran was used as control.

### FTIR analysis

The molecular characteristic of chitosan, CSNPs, PCL, and final scaffold was investigated with Fourier Transform Infrared (FTIR) spectroscopy in the wavenumber range of 4000 cm^-1^ to 400 cm^-1^ with 4 cm^-1^ resolutions (Bomem MB-Series FT-IR Spectrophotometer, USA).

### Measurement of swelling and degradation rate

The swelling percentage of scaffolds was measured through immersing the samples in the PBS. The pieces of the scaffolds were soaked in PBS at room temperature for 24 h. After that, the excess PBS was absorbed using a filter paper. The swelling capacity was calculated by the Eq. (1):

1 $${\text{Swelling}}\,\left( \% \right) = \left( {W_{w} {-} \, W_{d} } \right)/W_{d} \times 100$$

where *W*_*d*_ is the initial dry weight and *W*_*w*_ is the weight of the sample after incubation in PBS.

In vitro degradation of the CSNP/chitosan/PCL blend scaffolds was also tested by immersing the pieces of the scaffolds in PBS (pH = 7.4) at 37 °C and monitored for 21 days. The samples were recovered from the PBS at the determined times and dried in the oven at 37 °C. Degradation of samples was calculated using Eq. (2) after defined time intervals:

2 $${\text{Weight}}\,{\text{Degradation}}\,\left( \% \right) = \left( {W_{{1 \, {-}}} W_{2} } \right)/W_{1} \times 100$$

*W*_*1*_ and *W*_*2*_ are the sample weights before and after in vitro degradation, respectively.

### HCECs isolation and culture

The stripped Descemet’s scaffolds containing HCECs obtained from EYE Bank of I.R. Iran were washed with sterile PBS and then incubated in crude collagenase (1 mg/ml) for 1 h at 37 °C while shaking gently to isolate the HCECs. The viability of enzymatically isolated HCECs was evaluated using trypan blue staining. After that, the harvested cells were cultured on culture plates in DMEM/F12 medium containing 10% FBS and 1% penicillin/streptomycin. 5 µM of Rho-associated kinase (ROCK) inhibitor Y-27632 (Abcam, ab120129) was added to the medium for enhancement of HCECs adhesion and proliferation^31^ and incubated at 37 °C, 5% CO_2_ and 95% humidity. The medium was replaced every 2–3 days. When the HCECs became confluent, they were detached from culture plates and prepared for culture on the CSNP/chitosan/PCL composite scaffolds. All humane cellular experiments were performed according to the criteria of the ethics committee of Shahid Beheshti University of Medical Sciences.

### Corneal endothelial cells adhesion assay

The scaffolds were cut into circular pieces with equal size, neutralized with NaOH 1 N for 1 h and then carefully washed with distilled water. Subsequently, the pieces were sterilized by incubation in 70% ethanol for 2 h and washed three times with sterile PBS. Next, the sterilized scaffolds were flattened and immobilized in 24-well plates using Pyrex rings and soaked in DMEM/F12. After 24 h incubation of the scaffolds, the medium was changed and the HCECs were seeded at the density of 4 × 10^4^ cell/well in the fresh DMEM/F12 containing 1% penicillin/streptomycin, 10% FBS and 5 µM of Rho-associated kinase (ROCK) inhibitor Y-27632 and incubated for next 24 h in 37 °C by 5% CO_2_ and 95% humidity. Then, the non-adherent cells were washed with PBS and the attached cells were detached by 0.05% trypsin and counted using a Neubauer counting chamber.

### Hematoxylin and eosin staining

Sections of scaffolds with attached HCECs were fixed by 4% paraformaldehyde for 20 min at 4 °C and embedded in paraffin blocks using routine standard methods, followed by hematoxylin & eosin (H&E) staining. The stained sections were then evaluated by light microscopy.

### Evaluation of HCECs proliferation

The MTT assay was done to assess the cell proliferation of fabricated composite scaffolds. The scaffolds were prepared as previously described in “Corneal endothelial cells adhesion assay” section and flattened and fixed in 24-well plates in DMEM/F12 for 24 h. Then, the medium was changed with fresh DMEM/F12 medium containing 10% fetal bovine serum (FBS), 1% penicillin/streptomycin and 5 µM of Rho-associated kinase (ROCK) inhibitor Y-27632. HCECs were seeded at the density of 1 × 10^5^ cells per well. The cells cultured on culture plate in the same medium were considered as the control and in each experiment, the cell-free cultures were used as the blank. MTT assay was carried out 1, 2, 3, and 7 days after cell seeding. Culture medium was changed by 450 µL fresh medium and 50 µL MTT solution was added to each well and the plates were incubated in 37 °C for 4 h. Then, the medium was gently removed and the formazan crystals were dissolved using 500 µL dimethyl sulfoxide (DMSO). The absorbance of formazan solutions was measured at 570 nm using a microplate reader (ELx 800, BioTek, USA).

### Flow cytometry

The phenotypic maintenance of cultured HCECs during culture period was evaluated through investigating the expression of specific surface markers at 3 days and 10 days after culture. The cells were collected from culture plates by 0.05% trypsin and suspended in DMEM containing 1% FBS at the density of 2 × 10^5^ cell/ml. The cells were incubated with PECy5-conjugated anti-human CD44 (1:100; Abcam) and FITC-conjugated anti-human CD166 (1:90; Abcam) for 2 h at 4 °C. After washing by PBS containing 1% FBS, the cells were analyzed using the BD FACS Canto II (BD FACS Canto II Cell Analyzer, BD Biosciences).

### Electron microscopy

The adhesion of the HCECs was also investigated by scanning electron microscopy (SEM) for cells cultured on membranes and culture plates (control). The samples were fixed by incubation in 4% paraformaldehyde for 20 min at 4 °C and dehydrated through immersing them in ascending concentrations of ethanol (30, 50, 70, 80, 90, and 100%) each for 5 min. After that, the samples were completely dried in a desiccator for 30 min and gold sputter-coated according to standard procedure and finally analyzed with scanning electron microscopy (TESCAN-VEGA-II).

### Statistical analysis

All quantitative experiments were done triplicate and data were presented as mean ± standard deviation (SD). The data were analyzed using one-way ANOVA followed by Tukey’s multiple comparison tests. *P* < 0.05 was considered statistically significant.

## Results

### Characterization of chitosan nanoparticles

**Figure 1:**
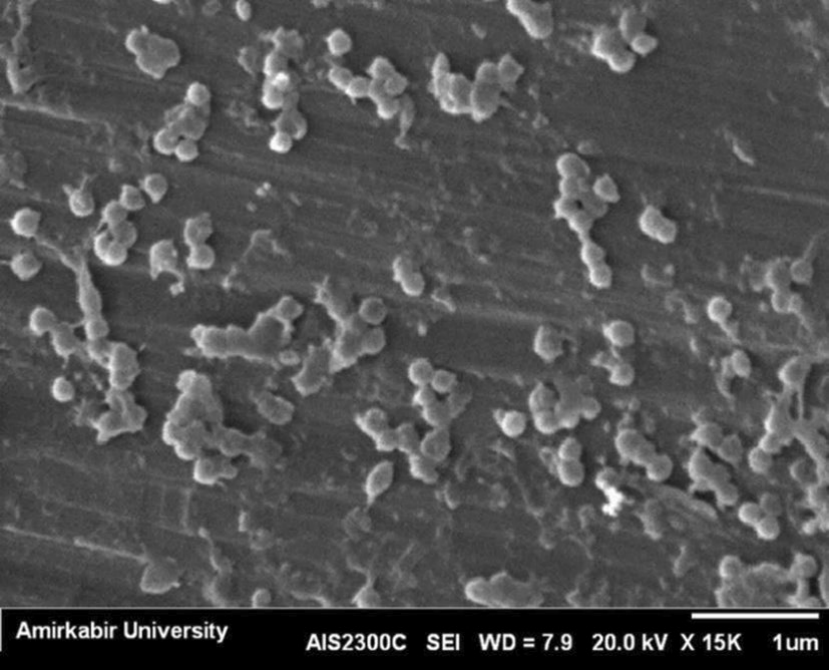
Representative SEM image of chitosan nanoparticles.

Evaluating the size of purified CSNPs and CS-PCL loaded CSNPs by DLS analysis showed a narrow size distribution with the mean of 130 nm for purified CSNPs and 176 nm for loaded CSNPs. PDI value of purified CSNPs and CS-PCL loaded CSNPs were 0.21 and 0.19, respectively (Table 1). In addition, SEM imaging of CSNPs revealed an approximately uniform size and spherical form of nanoparticles (Fig. 1).

**Table 1:** CSNPs size properties evaluated by DLS.

Sample name	Diameter (nm) Mean ± SD	Polydispersity index Mean ± SD	Number of particle batches
Purified CSNP	130 ± 35.7	0.21 ± 0.043	3
CSNPs dispersed in CS-PCL solution	176 ± 27.6	0.19 ± 0.038	3

### Macroscopic properties of composite scaffolds

**Figure 2:**
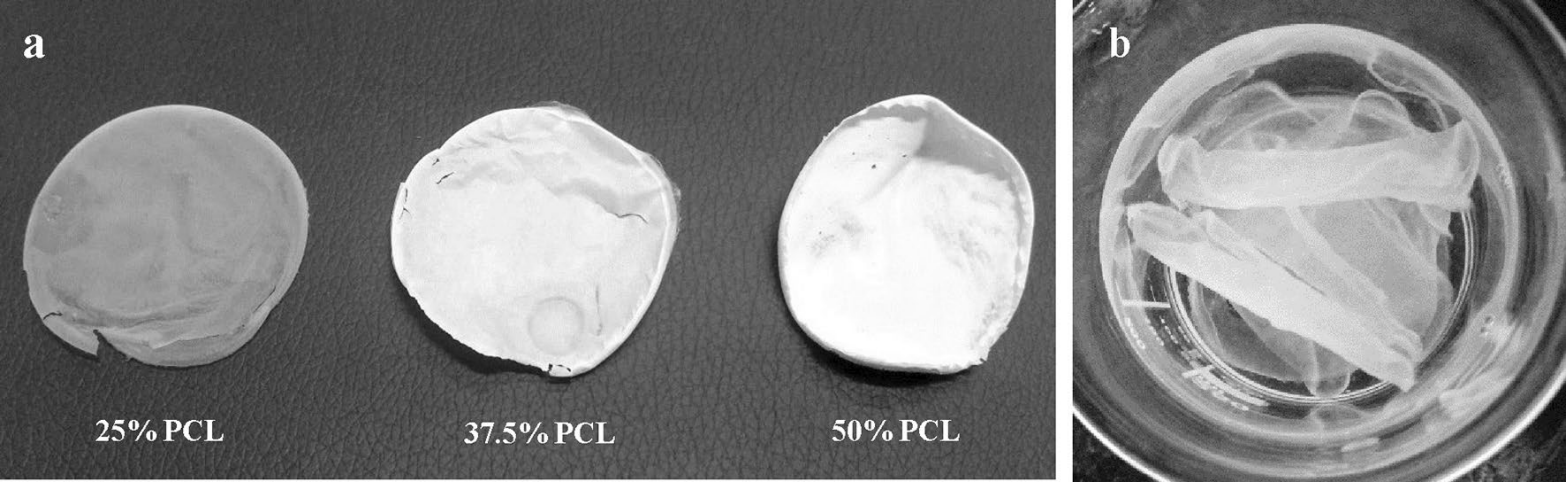
Macroscopic photographs of: (**a**) Dried composite membranes, visual transparency of the membrane decreased by increasing the PCL content. (**b**) CSNP/PCL 50/25 membranes became much more transparent and flexible by immersing in PBS compared with dried membranes. CSNP: chitosan nanoparticle, PCL: polycaprolactone.

The scaffolds were made through solvent casting and subsequent drying in an oven at 37 °C. All scaffolds were approximately uniform, although, the visual transparency decreased macroscopically by increasing the PCL content (Fig. 2a). Furthermore, in dry state, the scaffolds were fragile which became completely flexible and easy to handle after brief crosslinking and immersing in the PBS (Fig. 2b).

### Surface wettability

The results of water contact angle measurement have been represented in Table 2. There is an inverse relation between surface wettability and contact angle, thus, based on data, by increasing the ratio of CSNPs/PCL in the composites, the wettability of scaffolds was increased.

**Table 2:** Surface wettability of the composite scaffolds.

The composite samples^a^	WCA
CSNP/PCL 25/50	51° ± 2
CSNP/PCL 37.5/37.5	42° ± 2
CSNP/PCL 50/25	23° ± 1

^a^All samples had a constant concentration of chitosan (25% w/w), n = 6.

### Evaluation of light transmittance

**Figure 3:**
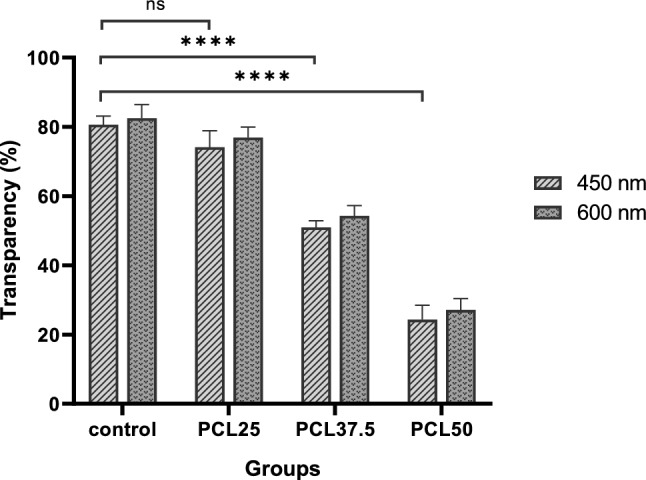
The effect of PCL content on light transmittance of the composite membranes. Increasing the PCL ratio in the composite led to increase the membrane opaque. All investigations were done triplicate (n = 3) under wet condition. Data was represented as mean ± SD (****P < 0.0001).

Measurement of transparency in the wavelength of 450 nm and 600 nm showed that between all composites, CSNP/PCL 50/25 scaffolds have the highest degree of transparency. As shown in Fig. 3, the transparency of acellular stroma is about 80% at 450 nm which has no significant difference with CSNP/PCL 50/25 scaffolds (about 74%). The clarity of scaffolds decreased by reducing the CSNP/PCL ratio. Both CSNP/PCL 37.5/37.5 and CSNP/PCL 25/50 scaffolds are significantly more opaque than control with 51% and 24% transparency, respectively (both *P* < 0.0001). The scaffold transparency is a critical feature for corneal regeneration; therefore, based on these results, we only used the CSNP/PCL 50/25 for subsequent analysis.

### FT-IR analysis

**Figure 4:**
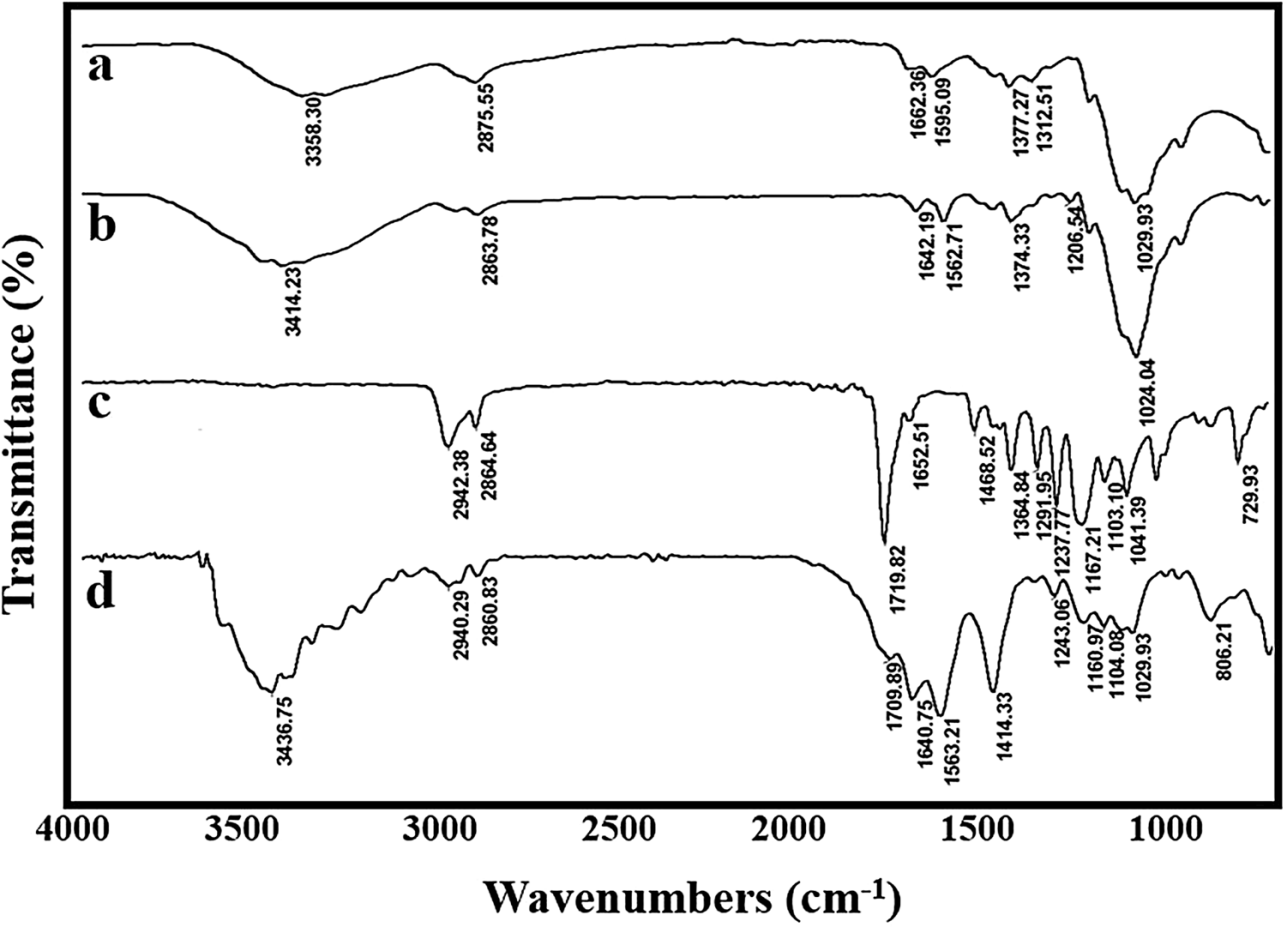
FT-IR spectra of the samples: chitosan (**a**), CSNPs (**b**), PCL (**c**), and CSNPs/PCL 50/25 composite membrane (**d**).

The chemical structure of samples was evaluated by FTIR spectroscopy analysis. As shown in Fig. 4a, the typical peaks of chitosan appeared at 3358 cm^−1^, 2875 cm^−1^, 1662 cm^−1^, 1595 cm^−1^, and 1029 cm^−1^ were related to the stretching vibrations of the –NH_2_ and –OH groups, –CH– and –CH_2_–, amide I, amide II, and C–O, respectively^32^. Compared with chitosan, the NH_2_ and –OH peak of the CSNPs became sharper and shifted to a higher wavenumber (3414 cm^−1^) which exhibited that the hydrogen bonding is enhanced. Also, the new peak appears at 1206 cm^−1^ due to the stretching vibration of electrostatic bonds of P=O (Fig. 4b). Moreover, the NH_2_ peak shifted to lower wavenumber (1562 cm^−1^) which confirmed the strong crosslinking between amine and phosphate groups from chitosan and TPP, respectively^33,34^. In the spectrum of the PCL (Fig. 4c), several characteristic peaks were observed at 2942 cm^−1^ and 2864 cm^−1^, 1719 cm^−1^, and 1167 cm^−1^. These peaks are attributed to stretching vibrations of asymmetric and symmetric -CH_2_-, carbonyl group, and symmetric –C–O–C, respectively^32^. The FT-IR spectrum of the scaffold (Fig. 4d) exhibits a characteristic band of both CSNPs and PCL i.e. –NH_2_, –CH_2_–, C=O, P=O, and C–O, which indicated the uniform chemical structure of the prepared scaffold.

### Swelling and degradation behavior

**Figure 5:**
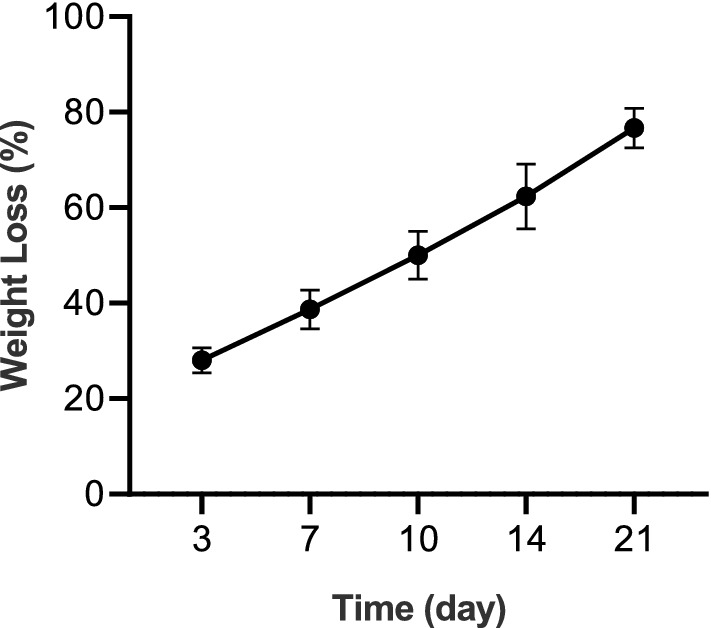
Degradation profile of CSNP/PCL 50/25 membrane during 21 days. All investigations were done triplicate (n = 3) in PBS (pH = 7.4) at 37 °C. Data was represented as mean ± SD.

The measured swelling rate of CSNP/PCL 50/25 scaffold was 61.7% ± 2.3%, after 24 h incubation in PBS at room temperature. Degradation properties of the scaffold in the PBS have been shown in Fig. 5. The assay was done at 37 °C in defined time intervals. About 28% and 39% of the scaffold weight were lost at days 3 and 7, respectively. The slope of degradation rate was approximately constant during all 21 days and at the end, about 76% of the scaffold was degraded. The shape of the scaffolds was nearly preserved throughout the degradation assessment.

### HCECs isolation and culture

**Figure 6:**
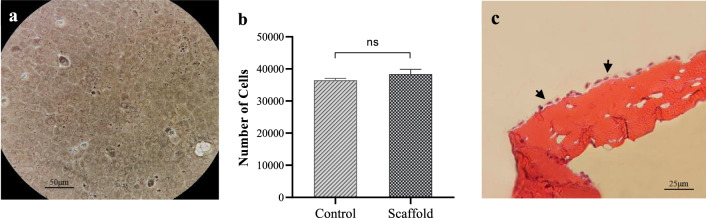
(**a**) Primary cultured HCECs on tissue culture plate after reaching to confluence. (**b**) Quantitative evaluation of the HCECs count attached to the culture dish (as a control) and CSNP/PCL 50/25 membrane. (**c**) H&E staining of continuous HCECs adhered on the scaffold (arrows).

Trypan blue staining of HCECs showed that about 96% of isolated cells were alive. The cell cultured in the presence of Rho-associated kinase (ROCK) inhibitor Y-27632 reached the desired confluence within 10 days. Microscopic observation of confluent cells showed a compact layer of cells with HCECs specific polygonal morphology (Fig. 6a).

### Cell adhesion

Cell counting of the adherent cells seeded on the scaffolds revealed that the number of attached HCECs in the control and scaffold groups was not significantly different after 24 h (Fig. 6b). H&E staining results also showed a continuous monolayer of HCECs on the surface of the film (Fig. 6c).

### Cell proliferation and cytotoxicity assay

**Figure 7:**
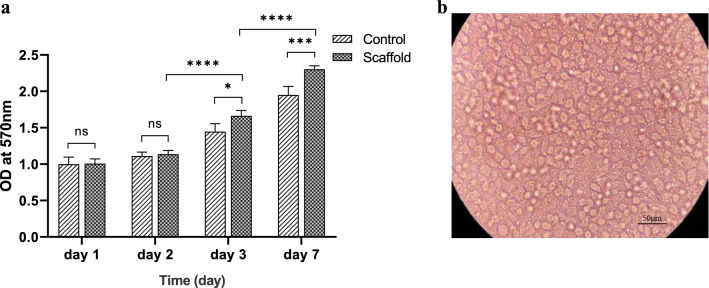
(**a**) Viability and proliferation of HCECs cultured on CSNP/PCL 50/25 membrane compared with control group. Data was represented as mean ± SD. (**b**) Light microscopy of the confluent HCECs cultured on the CSNPs/PCL 50/25 membrane at day 7.

Cell viability and proliferation on CSNP/PCL 50/25 membranes were assessed by MTT assay at 1, 2, 3, and 7 days after culturing of the HCECs on the membranes. According to Fig. 7a, there is no significant difference between the viability of the HCECs cultured on cultured plates (control group) and the cells cultured on membranes (scaffold group) at days 1 and 2. At days 3 and 7, the viability of HCECs cultured on membranes was significantly more than the control group (*P* < 0.05 & *P* < 0.001, respectively). Moreover, the viability of HCECs cultured on membranes significantly increased after day 3 compared with those of day 2 (*P* < 0.0001), which shows that the cells proliferated after day 3.

### Flow cytometry

**Figure 8:**
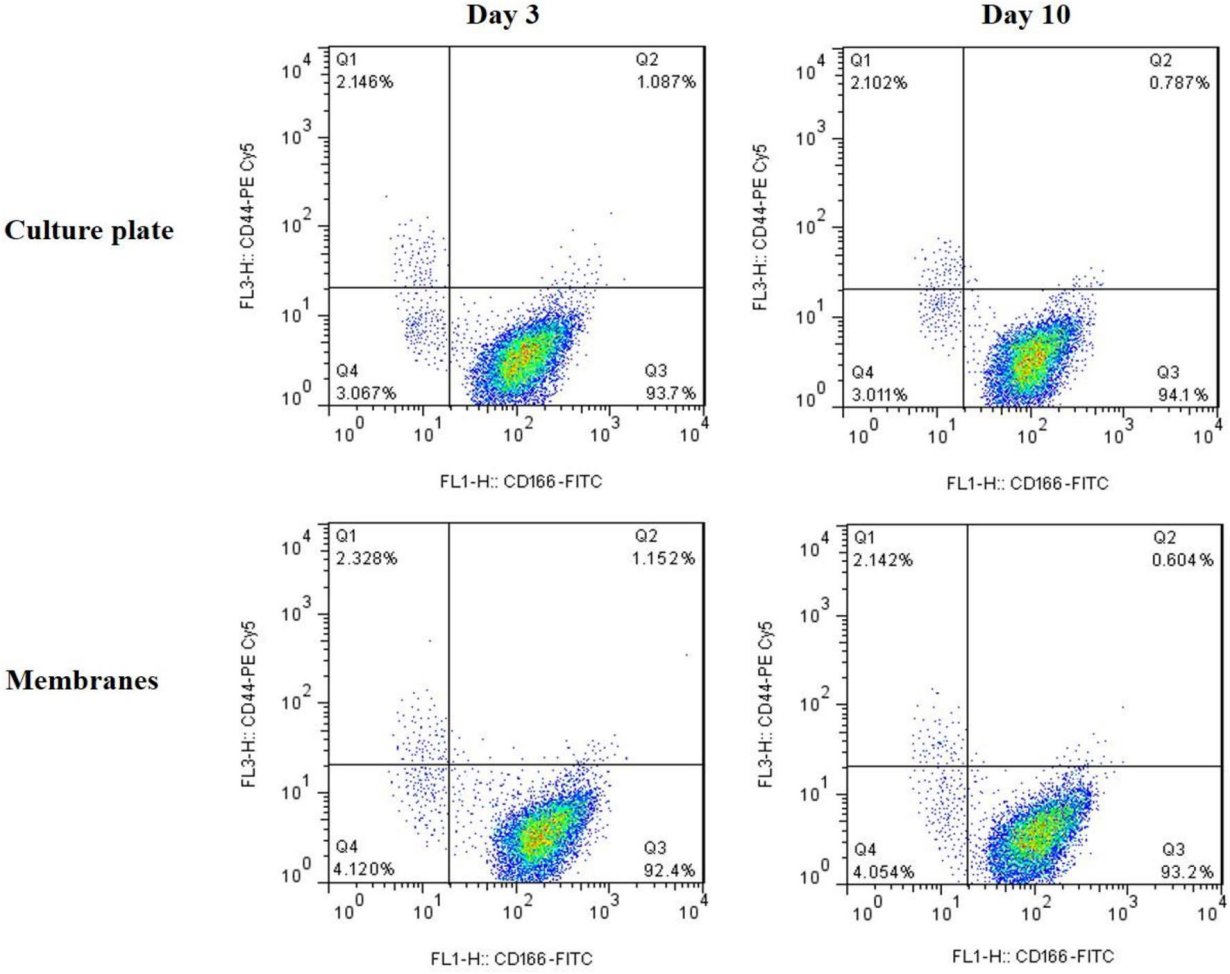
Flow cytometer analysis of CD166 and CD44 expression during culture period by HCECs cultured on culture plate (control) and CSNPs/PCL 50/25 membrane after 3 and 10 days.

Phenotypic examination of HCECs by flow cytometry showed that the cell phenotype marker was maintained during the culture period both in the cells cultured on membranes and those of culture plates (control) (Fig. 8). By simultaneous detection of two surface markers, the results showed a high expression of CD166, while CD44 was poorly expressed in all groups. At day 3, the expression rate of CD166 in control and membrane groups were 93.7% ± 3.1% and 92.4% ± 2.4%, respectively. In contrast, the CD44 expression of both groups was very low (2.146% ± 0.45% for control and 2.328% ± 0.39% for membrane group). After 10 days from culture, the expression of markers was approximately constant. As shown in Fig. 8, the rate of CD166 expression in control and membrane groups was 94.1% ± 2.7% and 93.2% ± 1.8%; while, the CD44 expression was 2.102% ± 0.63% and 2.142% ± 0.57%, respectively.

### Electron microscopy

**Figure 9:**
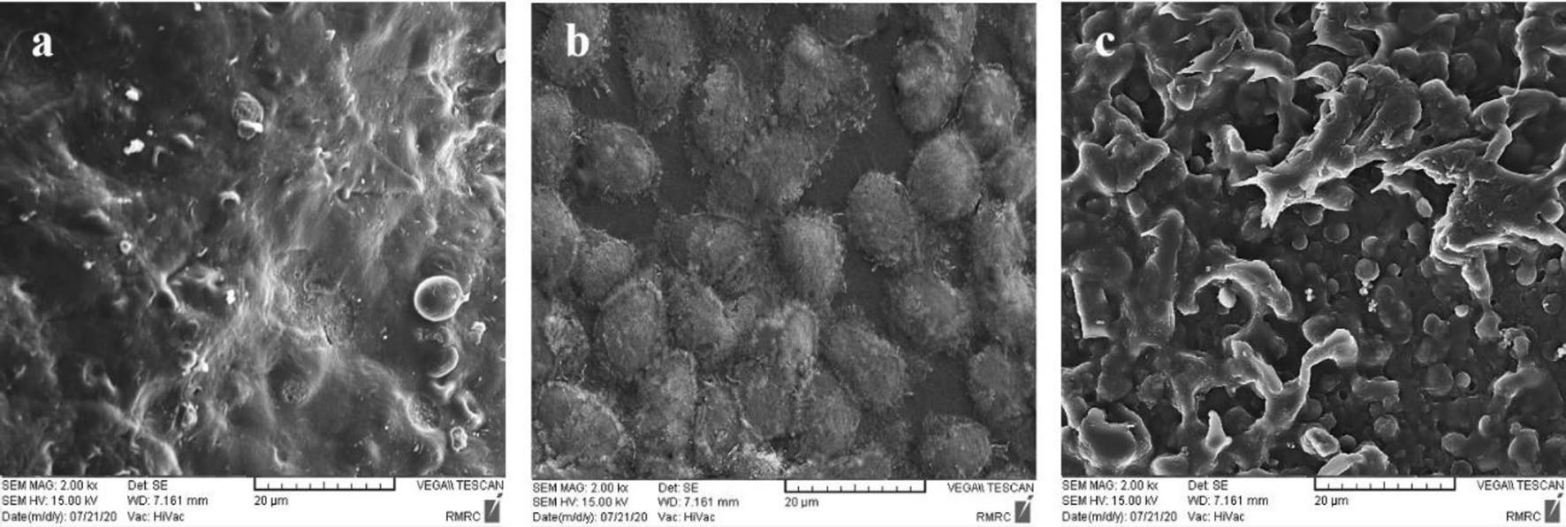
Representative SEM images of: (**a**) cell-free CSNP/PCL 50/25 membrane, (**b**) HCECs cultured on culture plate as control for 5 days, (**c**) HCECs cultured on CSNP/PCL 50/25 for 5 days.

SEM imaging of the cell-free scaffold exhibited that the surface of constructed membrane was rough to support the cultured HCECs (Fig. 9a). The SEM imaging of HCECs cultured on culture plate showed the high density of the round cells (Fig. 9b). Moreover, the HCECs were appropriately attached and flatted on the designed membrane after 5 days which represents the good interaction between cells and surface (Fig. 9c).

## Discussion

Several research groups have recently focused on finding new strategies for corneal endothelial regeneration instead of endothelial keratoplasty (EK) because of the global shortage of donors. Application of artificial corneal endothelial cell sheets designed and fabricated through tissue engineering approaches is an alternative therapy for corneal endothelial disorders^35^. Combination of two or more polymers is one of the surface modification methods which results in the improvement of scaffold features which enhance the cells-surface interactions and are very important issues for biomedical applications of a scaffold^36,37^. In this study, chitosan and PCL were used to fabricate the scaffold for culture of human corneal endothelial cells. In addition to numerous literature which have previously investigated chitosan safety and biocompatibility, we chose it based on our targeted tissue.

To the best of our knowledge, there is not much evidence for the use of nanoparticles in corneal endothelial tissue engineering. Nanoparticles are used in tissue engineering to mimic the natural extracellular matrix because they increase the possibility of controlling scaffold features, such as biomechanical and biological properties, especially control release of bioactive molecules^38^. The result of this study showed that nanoparticles increase surface wettability which in turn enhances the cell-surface interactions such as cell adhesion, migration, and proliferation.

The mechanical and biochemical properties of PCL/chitosan blends have been previously studied^39–41^. Furthermore, the application of the PCL/chitosan films for corneal endothelial regeneration has been investigated by Wang et al. and Young et al. According to the results of these studies, the viability of corneal endothelial cells (CECs) cultured on pure chitosan scaffolds was gradually decreased, thus, PCL was added to chitosan to improve the cell adhesion^42,43^. However, PCL has some drawbacks such as hydrophobicity, slow rate of degradation, and pH disruption^40^. In the current study, the chitosan nanoparticles were incorporated into the PCL/chitosan composite to enhance the surface properties. Compared to previous studies, our results showed that at the same proportion of PCL in the composite, using chitosan nanoparticles leads to increasing the cell proliferation over 7 days. It has been shown that application of nanoparticles in scaffolds results in improvement of surface area and surface energy through increasing surface wettability^44^. The results show that the increasing CSNP/PCL ratio in the composites significantly decreased the water contact angle. The WCA of a composite is considered as an indicator of surface wettability and hydrophilicity, thus, these results confirmed that incorporation of CSNPs lead to enhancement of the surface wettability that subsequently improved cell and surface interactions^45^.

Obtaining the appropriate particle size and small size distribution of CSNPs was very important because the uniformity of particle could affect the uniformity and clarity of the scaffold^46^. Furthermore, the uniform size of nanoparticles expresses that they have similar physicochemical properties^47^. The narrow size distribution of nanoparticles obtained from DLS analysis of purified and CS-PCL blend loaded CSNPs showed that this aim was achieved. In addition, based on the FTIR investigation, presence of characteristic peaks of chitosan, PCL, and CSNP indicated that all components were effectively bound together which resulted in construction of a uniform membrane.

The clarity of the scaffold used for corneal regeneration is one of the most important features for corneal tissue engineering^48^. Also, the scaffold transparency makes it possible for in vitro and in vivo microscopic tracking of adhesion, proliferation, and morphology of the cells. In this study, a clear film was produced which could be applied in corneal endothelium regeneration due to its good transparency. The clarity of CSNP/PCL 50/25 was approximately similar to the acellular human corneal stroma as control. It seems that the rate of transparency depends on the content of PCL in the composites. Reducing the concentration of PCL not only results in more transparent membrane but also reduces the PCL disadvantages^41^. Our data of transparency was consistent with a previous study that showed increasing the PCL concentration in the PCL/chitosan blends results in decrease membrane transparency^39^. Based on our pilot study, we found that the concentration of CSNP has no remarkable impact on the transparency of chitosan films (data not shown).

From the results of the study, the water content of our developed composite was about 64% and the fragile membranes became well flexible after swelling. The swelling capacity of the scaffold is an important feature which allows the exchange of fluids, nutrients, and metabolites through the scaffold as well as improves the interaction of cell and surface. However, it is necessary to control the rate of swelling under physiological condition because it may cause the fast degradation of the scaffold^49^.

Based on the results, the designed membrane had a proper degradation rate. Furthermore*, *in vitro culture of HCECs on the scaffold showed that after one week the cells became completely confluent (Fig. 7b). So, it can be estimated that the rate of scaffold degradation is proper for the formation of new corneal endothelium.

Composite compatibility and cell proliferation were evaluated via in vitro MTT assay. Only CSNP/PCL 50/25 composite film was selected for cell culturing and investigation of biocompatibility because of its good transparency and surface wettability. The viability of the HCECs on culture plate (control) and composite membranes were not significantly different at first 48 h. But, it can be seen that after 3 days the cell viability was increased that it results from the conditioning of the cells in the environment which lead to cell proliferation. The viability of HCECs on the film remarkably increased during the culture time compared with control groups. Surprisingly, the number of HCECs cultured on membrane significantly increased after 2 days. These data demonstrate that the fabricated scaffold not only is not cytotoxic but also is so biocompatible which provides an appropriate microenvironment for cell adhesion and proliferation.

The adhesion of corneal endothelial cells on the scaffold was quantitatively and qualitatively examined. Cell counts for adherent cells showed that approximately 93% of the cells attached to the scaffold which was not significantly different from the control group (88%). This result provides further evidence for good cell adhesion capability of the CSNP/PCL 50/25 membranes. In the natural cornea, the endothelial cells arranged in a compact monolayer of cells on the Descemet’s membrane^50^. The results of H&E staining showed that the cultured corneal endothelial cells form a continuous monolayer of cells on the surface of membrane similar to what is seen in the natural corneal endothelium. The SEM results of HCECs show that the HCECs are firmly attached to the surface of the membrane and well flatted and connected to each other. This reveals that our constructed membrane could appropriately support the HCECs for attachment, survival and proliferation. These results verify the result of H&E staining and light microscopy imaging.

Many cell surface markers are used to perform HCECs phenotyping, including CD166, CD105, CD44, CD26, CD24. These markers are phenotypic indicators for HCECs. The cells which were positive for CD166 and negative for CD105, CD44, CD26, CD24 were considered as efficient HCECs for therapeutic application^51,52^. The results of flow cytometry revealed that our cultured HCECs were strongly positive for CD166 and negative for CD44. These outcomes indicate that the phenotype of HCECs was maintained during culture time.

The developed scaffold seems to be suitable for use in corneal endothelium tissue engineering in terms of transparency, surface behaviors, and biocompatibility; however, further studies are needed to investigate the implantation of this scaffold in animal models. In addition, loading the bioactive molecules such as specific growth factors on CSNPs might be helpful for HCECs adhesion, proliferation, and functionality.

## Conclusion

We found that the concentration of PCL was inversely affecting the transparency of the films. Moreover, the results indicate that the incorporation of CSNP into PCL/chitosan films improved some specific characteristics of the scaffolds such as biocompatibility and surface properties, while preserved the transparency as a pivotal feature of a corneal scaffold. There is no significant difference between the transparency of the final scaffold (CSNP/PCL 50/25) and acellular corneal stroma, as a control. Additionally, MTT assay, H&E staining, and SEM imaging showed that cultured HCECs properly attached to the membranes and survived and proliferated appropriately during the culture period.

## Data Availability

The data that supports the findings of this study are available within the article.
